# Infectious keratitis after corneal crosslinking for keratoconus caused by levofloxacin-resistant microorganisms

**DOI:** 10.1186/s12886-021-02081-4

**Published:** 2021-08-31

**Authors:** Naoko Kato, Takeshi Ide, Hidenaga Kobashi, Ikuko Toda

**Affiliations:** 1 Minamiaoyama Eye Clinic, Kitaaoyama 3-3-11, Minato-ku, 107-0061 Tokyo, Japan; 2 Department of Ophthalmology, School of Medicine, Keio University, Tokyo, Japan; 3 Tokyo Vision Eye Clinic Asagaya, Tokyo, Japan

**Keywords:** Infectious keratitis, Corneal crosslinking, Levofloxacin-resistant, Methicillin- resistant *Staphylococcus aureus*, Methicillin-sensitive *Staphylococcus aureus*

## Abstract

**Introduction:**

We present seven cases of infectious keratitis after corneal crosslinking (CXL) to attenuate keratoconus progression.

**Methods:**

Of 524 consecutive patients who underwent CXL, 7 cases (4 males and 3 females; 21.5 ± 7.1 years) developed postoperative infectious keratitis were retrospectively reviewed. CXL was performed using the Dresden protocol or an accelerated protocol involving epithelial removal.

**Results:**

All cases appeared normal on the day after surgery, but subsequently developed eye pain, blurred vision, corneal infiltration, inflammation of the anterior chamber, and ciliary injection on day 2 or 3. Methicillin-resistant *Staphylococcus aureus* was cultured from two eyes, methicillin-sensitive *Staphylococcus aureus* from two eyes, and *Streptococcus pneumoniae* from one eye. All detected bacteria were resistant to levofloxacin (LVFX). Five of the seven cases, especially four of the five severe cases with hypopyon, had a history of atopic dermatitis. All cases were observed after 2015.

**Conclusions:**

Infectious keratitis after CXL caused by microbes resistant to LVFX is increasing. In addition to careful postoperative observation of the cornea, preoperative evaluation of bacteria within the conjunctival sac evident on nasal swab cultures may be useful to identify potentially problematic microbes and inform the selection of appropriate antibiotics.

## Introduction

Corneal crosslinking (CXL) attenuates keratoconus progression, as first reported by Wollensak et al. in 2003 [1]. Progression was halted in 90–95 % of cases in clinical trials [2–5]. However, several rare complications have been described, including delayed epithelial healing [6, 7], stromal melting [7, 8], sterile infiltration [8–10], early infectious keratitis [11–21], stromal hazing and demarcation after several weeks [9], deep stromal scarring [22], failure [9],  and progressive flattening [23, 24]. Infectious keratitis is the most concerning complication; scarring and irregular astigmatism may develop even after infection control, thus impairing the vision of young patients. We first used CXL to treat keratoconus 13 years ago, and encountered no case of infectious keratitis up to 2015. Sporadic cases were noted from 2016 onward (Table 1); these cases are summarized in this paper, including the possible causes and preventative measures and treatments that we employ.

**Table 1:** Demographic of cases with infectious keratitis after CXL at Minamiaoyama Eye Clinic

	Number of cases with infection	Number of CXL	Frequency (%)
2007	0	3	0.0
2008	0	2	0.0
2009	0	10	0.0
2010	0	12	0.0
2011	0	8	0.0
2012	0	5	0.0
2013	0	4	0.0
2014	0	45	0.0
2015	0	52	0.0
2016	2	46	4.4
2017	0	60	0
2018	1	92	1.1
2019	3	134	2.2
2020	1	51	2.0
Total	7	524	1.3

## Patients

From February 2007 to May 2020, 524 consecutively enrolled eyes of keratoconus patients, exhibiting pellucid marginal degeneration or keratectasia after laser in-situ keratomileusis, underwent CXL with epithelial debridement under topical anesthesia with application of oxybuprocaine hydrochloride (0.4 % w/v) eyedrops before each procedure. After the eye lid skin was delicately rubbed 2 times using a sterilized cotton swab soaked in the 10 % povidone-iodine solution, a lid speculum was inserted. The eye surface was rinsed with 20 ml of 5 ppm ozone water, followed by removal of the central corneal epithelium (diameter = 7.0–8.0 mm) using a blunt spatula or an excimer laser. Then, 0.1 % (w/v) isotonic riboflavin in dextran 20 % (w/v) solution was instilled every 2 min for 20 min. After confirming stromal riboflavin saturation via slit-lamp examination, the thinnest part of the corneal stroma was measured using an AL-3000 pachymeter (Tomey, Aichi, Japan). If the stromal thickness was < 400 μm, 0.1 % (w/v) hypotonic riboflavin or distilled water was instilled until the minimal thickness reached 400 μm. UV-A radiation was then delivered at 3.0 mW/cm^2^ for 30 min (KERA-X; Peschke, Huenenberg, Switzerland) or 18.0 mW/cm^2^ for 5 min (KXL; Avedro, Waltham, MA, USA). At the end of the procedure, a soft bandage contact lens was applied and a drop of levofloxacin (LVFX) instilled. Postoperative medications included LVFX and betamethasone (0.1 % w/v) eyedrops four times daily.

The study protocol was approved by the Internal Review Board of Minamiaoyama Eye Clinic. While this study was the retrospective observational study, the written informed consent was waived by the Internal Review Board of Minamiaoyama Eye Clinic. In all cases, the CXL was performed by experienced ophthalmologists.

The characteristics of the seven patients are summarized in Table 2: bacterial infections were proven or strongly suspected in all patients. Microbiological evaluations were performed for five patients; methicillin-resistant *Staphylococcus aureus* (MRSA) was cultured from two eyes, methicillin-sensitive *Staphylococcus aureus* from two eyes, and *Streptococcus pneumoniae* from one eye. All detected bacteria were resistant to LVFX. Four experienced surgeons performed the CXL for the seven cases, and there was no remarkable prevalence among individuals.

**Table 2:** Characteristics of cases with infectious keratitis after corneal crosslinking

Case	Age	Gender	AD	Year of surgery	Microbes	Sensitivity for antibiotics	Hypopyon	Clinical course	Antibiotics used for treatment	Others
1	21	M	+	2016	MRSA	R; LVFX, CTZ, EM S; VCM	+	Pain on day 3, healed with scar, eventual PKP	VCM	Ferrara ring implantation was performed simultaneously with CXL
2	33	F	-	2016	St pneumoniae	R; EM I; LVFX S; CTZ	-	Pain on day 2, Diagnosed on day 3	CTZ	
3	15	M	+	2018	MSSA	R; LVFX S; CTZ	+	Pain on day 2, Diagnosed on day 3	CTZ	
4	20	M	-	2019	Not tested	NA	+	Pain on day 2, Diagnosed on day 3	TOB, CP, CTZ	
5	25	F	+	2019	MSSA	R; LVFX, EM S; CTZ	+	Pain and visual disturbance on day 2 Corneal perforation on day 3	TOB, CTZ	
6	16	M	+	2019	Not tested	NA	-	Diagnosed on day 3	TOB, CP, CTZ	
7	16	F	+	2020	MRSA	R; LVFX, CTZ	+	Pain and diagnosed on day 2	VCM	

*AD* atopic dermatitis; *R* resistant; *S* sensitive; *PKP* penetrating keratoplasty; *LVFX* levofloxacin; *CTZ* ceftazidime; *EM* erythromycin; *VCM* vancomycin; *TOB* tobramycin; *CXL* corneal crosslinking, *MRSA* methicillin-resistant Staphylococcus aureus; *MSSA* methicillin‐sensitive Staphylococcus aureus; *St pneumoniae* Streptococcus pneumoniae

Five patients (1, 3, 4, 5 and 7) exhibited severe infectious keratitis with hypopyons. One patient (Case 1) underwent implantation of a pair of Ferrara rings contemporaneously with CXL. All five patients with severe keratitis appeared normal on the day after surgery, but complained of eye pain on day 2 or 3. All patients exhibited corneal epithelial defects accompanied by infiltration, ciliary injection, and a hypopyon on day 3. The infections were controlled by antibiotics, and the corneas healed but with stromal scarring. In the two patients without hypopyons, two (Cases 2 and 6) complained of pain on day 2 or 3 and were diagnosed with infectious keratitis on day 3. These two patients recovered quickly after application of topical antibiotics, without stromal scarring.

## Discussion

We encountered 7 cases (among 524 cases; rate of 1.34 %) of infectious keratitis after CXL to treat keratoconus, all within the past 5 years. MRSA and MRSA resistant to LVFX were the most frequent causative microbes, followed by *S. pneumoniae* (also resistant to LVFX). The frequency of infectious keratitis after CXL has been reported by several clinicians; the microbes involved were gram-positive bacteria (*Staphylococcus epidermidis*[21], *S. aureus*[11–16] and *Streptococcus salivarius* and/or *S. oralis*[17, 18]); gram-negative bacteria (*Escherichia coli*[19] and *Pseudomonas aeruginosa*[20]), herpes virus[25], and a fungus and *Acanthamoeba*[26]. Many microbes were resistant to the new quinolone antibiotics, similar to our findings.

As stated above, post-CXL infections were not observed until 2016 in our institute. In the 9 years from 2007 to 2015, we performed CXL on 141 eyes and experienced no case of infection. After the first case in 2016, the incidence increased to 3 of 134 eyes (2.2 %) in 2019 (Table 1).

We used ofloxacin eyedrops until 2000, and later LVFX eyedrops from 2001 still now, as preventative therapies after CXL, PRK, and PTK. The new quinolone antibiotics, such as LVFX, gatifloxacin, and moxifloxacin, have widely been used to prevent infection after various ophthalmological surgeries. The trends in antibiotic resistance among ocular microorganisms have been investigated. Asbell et al. showed that antibiotic resistance was prevalent among staphylococcal isolates, but only a few small changes were observed from 2009 to 2018 in the USA. [27, 28] Deguchi et al. found that the prevalence of MRSA and methicillin-resistant-coagulase-negative staphylococci (MR-CNS) decreased significantly from 2005 to 2014 in Japan, but over 50 % of *Corynebacterium*isolates remained resistant to LVFX[29]. Kamo et al. showed that the prevalence of LVFX-resistant MSSA increased significantly from 2008 to 2018, although the prevalence of MRSA did not increase. MR-CNS was not detected in 2008.[30, 31] Thus, microorganisms resistant to new quinolone antibiotics may have increased in recent years, raising the rate of postoperative infectious keratitis.

A history of atopic dermatitis may increase the risk of post-CXL infection. Skin MRSA levels are elevated in patients with atopic dermatitis[13, 32, 33]. Five of the seven patients (71 %) in this study had been diagnosed with atopic dermatitis, including four of the five (80 %) with severe infectious keratitis accompanied by a hypopyon. Patients on long-term steroids or immunosuppressants to treat allergic disorders, such as vernal keratoconjunctivitis, bronchial asthma, and eczema, require special attention in terms of the evaluation of drug-resistant bacteria, especially those with atopic dermatitis. Although there were no healthcare workers among our patients, that group also requires special attention because they frequently come into contact with drug-resistant bacteria[33]..

In this study, all symptoms of infection became apparent on postoperative day 2 to 3; no symptoms were noted on day 1. Postoperative examinations should be performed on day 2 or 3; if suspicious symptoms are seen, immediate microbiological testing (including for drug sensitivity) is required. We recently began to preoperatively screen for microbes via nasal culture; the conjunctival sac is seldom culture-positive when patients do not develop conjunctivitis. Furthermore, we prescribe additional effective antibiotics, such as chloramphenicol, postoperatively, if some bacteria resistant to LVFX and/or other drugs were detected.

In conclusion, postoperative infectious keratitis after CXL is caused mainly by LVFX-resistant bacteria. As microbial resistance to the new quinolone antibiotics has increased recently, drug-resistant bacteria on the ocular surface, especially in patients with atopic dermatitis, should be screened for in patients undergoing CXL for keratoconus.

## Data Availability

All data generated or analyzed during this study are summarized in this published article as Table 2.
